# Association of PTPN22 1858T/T genotype with type 1 diabetes, Graves' disease but not with rheumatoid arthritis in Russian population

**DOI:** 10.18632/aging.100305

**Published:** 2011-04-06

**Authors:** Daria Zhebrun, Yulia Kudryashova, Alina Babenko, Alexei Maslyansky, Natalya Kunitskaya, Daria Popcova, Alexandra Klushina, Elena Grineva, Anna Kostareva, Evgeny Shlyakhto

**Affiliations:** ^1^ Laboratory of Immunology; Almazov Federal Heart, Blood and Endocrinology Center, Saint- Petersburg, Russia; ^2^ Institute of Endocrinology; Almazov Federal Heart, Blood and Endocrinology Center, Saint-Petersburg, Russia; ^3^ Department of rheumatology; Almazov Federal Heart, Blood and Endocrinology Center, Saint-Petersburg, Russia; ^4^ Institute of Molecular Biology and Genetics; Almazov Federal Heart, Blood and Endocrinology Center, Saint-Petersburg, Russia

**Keywords:** type 1 diabetes, Graves' disease, rheumatoid arthritis, protein tyrosine phosphatase nonreceptor 22, single-nucleotide polymorphism

## Abstract

The protein tyrosine phosphatase nonreceptor 22 gene (*PTPN22*) is an important negative regulator of signal transduction through the T-cell receptors (TCR). Recently a single-nucleotide polymorphism (SNP) 1858 C/T within this gene was shown to be a risk factor for several autoimmune diseases, such as rheumatoid arthritis (RA), Graves' Disease (GD), systemic lupus erythematosus (SLE), Wegener's granulomatosis (WG) and type 1 diabetes mellitus (T1D). The aim of this study was to analyze a possible association between 1858 C/T SNP and a number of autoimmune diseases, including RA, GD and T1D in Russian population. Patients with T1D, GD, RA and healthy controls were genotyped for the 1858 C/T SNP in *PTPN22* gene. We found a significant association between *PTPN22* 1858 C/T SNP and T1D and GD. 1858T/T genotype was observed more frequently in T1D and GD patients compared to control subjects. No such association was observed for RA. In concordance with a previous data establishing *PTPN22* 1858 C/T SNP association with several autoimmune diseases, our findings provide further evidence that the *PTPN22* gene may play an important role in the susceptibility to some autoimmune diseases.

## INTRODUCTION

Autoimmune diseases are a clinically diverse group of complex disorders such as type 1 diabetes mellitus (T1D), multiple sclerosis (MS), Graves' disease (GD), Crohn's disease, rheumatoid arthritis (RA), psoriasis, systemic lupus erythematosus (SLE), etc. In total, they affect 5-7% of the world population [1]. Autoimmune diseases occur as a result of the loss of physiological tolerance to self antigens and are characterized by persistent activation of immune cells, leading to tissue damage [2]. Besides other causes, these diseases have complex genetic component which involves many genes regulating the extent of immune response. Major histocompatibility complex (MHC), especially MHC class II, has the most potent genetic influence on susceptibility to autoimmune disease [1, 3]. Among multiple non-HLA genes involved in autoimmunity regulation, cytotoxic T-lymphocyte-associated antigen 4 (CTLA-4) [4-7], tumor necrosis factor (TNF) [3, 8] and protein tyrosine phosphatase nonreceptor 22 gene (PTPN22) are the most important.

The PTPN22 gene is located on chromosome 1p13 and encodes a lymphoid protein tyrosine phosphatase (LYP) that is important in negative control of T-cell activation and in T-cell development. PTPN22 belongs to a family of protein tyrosine phosphatases involved in preventing spontaneous T-cell activation by dephosphorylation and inactivation of T-cell receptor-associated kinases and their substrates [9, 10]. PTPN22 is specifically expressed in lymphocytes [11] and through formation of a complex with C-terminal Src Kinase (CSK) suppresses the downstream mediators of T-cell receptor signaling [12, 13]. Earlier, PTPN22 gene was found to be associated with susceptibility to T1D [14]. Later it was shown that several SNPs could potentially contribute to susceptibility to various autoimmune disorders. Among them 1858C/T SNP was the most stable, where T allele correlated with T1D, RA, SLE, GD, Addison disease, etc [15-20]. However, the data varied from population to population and some studies failed to demonstrate any associations between 1858C/T SNP and autoimmune diseases.

Using a conventional approach, we performed a case-control association study between 1858C/T polymorphism and type 1 diabetes, RA and Graves' disease in a Russian population.

## PATIENTS AND METHODS

### Patients

All patients and control subjects were from the North-West region of Russia. The study was performed after written inform consent and was approved by the Ethic committee from each institute and hospital participating in the study: Almazov Federal Heart, Blood and Endocrinology Centre, Saint-Petersburg Diabetology Center №1.

### T1D

The diagnosis was established before the age of 20 years, using following criteria (defined by the National Diabetes Data Group): blood glucose level, presence of ketosis at onset, low body mass index and the need for insulin treatment. The diagnosis of T1D was confirmed by the presence of at least one of the two major antibodies: GAD65 antibodies, and/or anti-tyrosine phosphate like molecule (ICA512) antibodies.

### RA

Patients with 1 to 34 year-lasting (average of 12,524±4,982 years) disease were included in the study, of which 87% were rheumatoid factor seropositive.

### GD

Patients with 1 to 15 year-lasting Graves' disease participated in the study. The diagnosis was confirmed by the presence of thyrotoxicosis (increased levels of free triiodothyronine, free thyroxine, thyroid-stimulating hormone (TSH) <0,1 mMU/l) associated with diffuse hyperfunctional goiter other autoimmune manifestations (Graves' ophthalmopathy) and/or increased level of TSH receptor antibodies.

Healthy control subjects had normal glucose tolerance and no family history of autoimmune diseases. Genomic DNA was isolated from the peripheral blood sample using a phenol-chloroform extraction method [21]. The study included 150 patients with T1D, 171 patients with GD, 121 patients with RA and 200 healthy donors.

Genotyping was performed by PCR-restriction fragment length polymorphism (RFLP) method. A region of the PTPN22 gene (218-bp) containing the C1858T SNP (R620W) was amplified by the PCR using genomic DNA. The sequence-specific primers used were: forward 5`-ACTGATAATGTTGCTTCAACGG-3` and reverse 5`-TCACCAGCTTCCTCAACCAC-3′[22]. Each 25-μl amplification reaction contained 2 μl (20-30 ng) of DNA, 2.5 μl 10 × PCR buffer, 200 μM dNTPs, 2 mM MgCl2, 200 nM of each primer and 2.5 U Taq polymerase. The PCR began with an initial denaturation at 94°C for 5 minutes, followed by 30 cycles consisting of 30 sec at 94°C, 60 sec at 62°C, and 60 sec at 72°C, and it ended with a final elongation step at 72°C for 5 minutes. Amplified products were digested using RsaI (SibEnzyme) overnight at 37°C. Digested products were electrophoresed on a 3% agarose gel and visualized by ethidium bromide. RsaI recognizes its target sequence only when the PTPN22 1858C allele is present. The 1858T allele is not digested and yields one fragment of 218 bp, while the 1858C allele is digested and yields two fragments of 176 bp and 46 bp.

### Statistical analysis

Data obtained for patients and controls were compared using Chi-square testing and Fisher exact test. All calculations were performed using STATISTICA 6.0 program.

## RESULTS

The genotype and allele frequencies are presented in Table 1. Distribution of genotypes was consistent with Hardy-Weinberg equilibrium in all groups of patients and in the control group.

The case-control study showed that the distribution of the PTPN22 1858C/T SNP genotypes differ significantly between T1D patients and healthy controls, and between GD patients and healthy controls. The disease-associated homozygous genotype T/T has an increased frequency in T1D and GD patients vs. control subjects: 7% and 4.1% vs. 1%, respectively (P<0.001, OR 7.84 (95% CI 1.71-35.90) for T1D and P<0.05, OR 4.23 (95% CI 0.87-20.62) for GD). However, there was no statistically significant association between T allele and these disorders. This observation can be partially explained both by the complexity of statistical analysis in small populations per se, and with low overall allele frequencies, as observed for PTPN22 gene polymorphism.

In the group of RA patients, no statistically significant difference was detected in a distribution of genotypes or alleles, comparing to the control subjects (Table 1).

**Table 1.** Frequency of PTPN22 1858C/T alleles and genotypesin patients and healthy controls^a^

Genotype or allele	Control (N = 200)	T1D (N = 150)	GD (N = 171)	RA (N = 121)
	N (%)			
T/T	2 (1)	11 (7)	7 (4)	2 (2)
C/T	66 (33)	40 (27)	49 (29)	38 (31)
C/C	132 (66)	99 (66)	115 (67)	81 (67)
P	-	<0.001	<0.05	NS ^c^
OR (95% CI) ^b^	-	7.84 (1.71-35.90)	4.23 (0.87-20.62)	3.50 (0.74-16.70)
Allele T	70 (17.5)	62 (20.5)	63 (18)	42 (17)
Allele C	330 (82.5)	238 (79.5)	279 (82)	200 (83)
P	-	NS	NS	NS

a values are the frequency (number) of genotypes/alleles. P values were tested using *x^2^* test for each patient group vs control group.

b the odds ratios (ORs) and 95% confidence intervals (95% CIs) are for carriage of TT versus CT+CC genotypes.

c not significant

## DISCUSSION

1858C/T SNP is located in the N-terminal proline-rich motif of PTPN22 and leads to the substitution of arginine with tryptophan at codon 620 in lymphoid protein tyrosine phosphatase. The 1858T allele (variant 620W) of PTPN22 is a gain-of-function form of the enzyme, thus it results in an increased inhibition of TCR-mediated signals [23]. In vitro studies showed that the disease-associated LYP variant 620W prevents the interaction of LYP with CSK [15]. The CSK-LYP complex normally suppresses T cell receptor signaling kinases Lck, ZAP-70 and FYN. However, in the presence of mutant 620W form of LYP, lack of CSK-LYP interaction leads to the uncontrolled T cell induction and results in the increase in overall reactivity of the immune system [24]. Disruption of mouse ortholog of the human PTPN22 gene resulted in elevated serum antibody levels and increased memory T cells number, which are able to aggravate any autoimmune process [19, 25].

Numerous studies revealed the correlation between 1858C/T SNP with RA [15, 16, 26-28], SLE [17, 29], T1D [14, 18, 30], GD [18, 19], Myasthenia Gravis [31], WG [32] etc. However, this association may be questionable for some of these diseases, such as SLE and RA, as a number of studies failed to demonstrate it [33, 34]. At the same time, no association with multiple sclerosis (MS) [35, 36], Crohn's disease [37-39], ulcerative colitis [39], psoriasis and psoriatic arthritis [36] was found so far.

In compliance with other authors we demonstrated a significant association between PTPN22 1858 C/T SNP and T1D and GD. As many autoimmune disorders T1D and GD are associated with abnormal activation of immune system, which results in damage of organs and tissues. T1D is characterized by autoimmune destruction of insulin-producing β-cells in the pancreas. GD is an organ-specific autoimmune thyroid disorder characterized by hyperthyroidism, various degrees of diffuse goiter and ophthalmopathy. Genetic susceptibility is important in the development of both disorders. Over the last three decades, the study of T1D has led the field in the identification of genes underlying complex multifactorial diseases. Besides HLA class 2 genes, gene encoding insulin, CTLA4 and interleukin-2 receptor alpha (IL2RA) genes, PTPN22 presumably contributes to T1D susceptibility [14, 18, 30, 40, 41]. GD was also reported to have a substantial genetic component [7, 19, 42].

In contrast to our data for T1D and GD, the association between PTPN22 1858C/T and RA could not be verified in our study. This may be explained by the variation in PTPN22 allele frequencies in different ethnic groups, as described by Mori et al [43]. According to the available data on PTPN22 allele frequencies distribution worldwide, the degree of the association between 1858C/T SNP and different autoimmune diseases is variable among ethnic populations, suggesting that 1858C/T SNP may be of little importance for RA in Russian population.

## Abbreviations

PTPN22: protein tyrosine phosphatase nonreceptor 22 gene
TCR: T-cell receptors
SNP: single-nucleotide polymorphism
RA: rheumatoid arthritis
GD: Graves' disease
SLE: systemic lupus erythematosus
WG: Wegener's granulomatosis
T1D: type 1 diabetes mellitus
RFLP: restriction fragment length polymorphism
MS: multiple sclerosis
MHC: major histocompatibility complex
CTLA-4: cytotoxic T-lymphocyte-associated antigen 4
TNF: tumor necrosis factor
LYP: lymphoid protein tyrosine phosphatase
CSK: C-terminal Src Kinase
OR: odds ratio
ZAP-70: zeta-chain (TCR) associated protein kinase 70kDa
IL2RA: interleukin-2 receptor alpha
TSH: thyroid-stimulating hormone
dNTP: deoxynucleotide triphosphate
